# Infection prevention and control measures during COVID‐19 from medical physics perspective: A single institution experience from China

**DOI:** 10.1002/acm2.12889

**Published:** 2020-04-28

**Authors:** Dingjie Li, Ru Liu, Shengtao Wei, Tian Li, Jing Cai, Hong Ge

**Affiliations:** ^1^ Department of Radiation Therapy Henan Cancer Hospital Zhengzhou Henan China; ^2^ Department of Health Technology and Informatics The Hong Kong Polytechnic University Hong Kong SAR China

The outbreak of novel coronavirus, named as severe acute respiratory syndrome coronavirus 2 (SARS‐CoV‐2), has caused a global pandemic. The number of cases who are infected with COVID‐19, the disease that SARS‐CoV‐2 causes, grew rapidly in China in late January and early February 2020, but has since plunged significantly owing to tremendous containment efforts by the government and health‐care professionals. The total number of confirmed cases outside China continue to escalate, especially in Europe and the USA, and on 24 April 2020, the number has reached 2.7 million. Current data suggest that COVID‐19 coronavirus is less deadly but more infectious than its related illnesses, such as severe acute respiratory syndrome (SARS) and Middle East respiratory syndrome (MERS). The spreading speed of SARS‐CoV‐2 is approximately four times that of SARS. The severity of COVID‐19 symptoms can range from very mild to severe and some people may be asymptomatic. These characteristics of COVID‐19 have made it extremely challenging for its prevention and control.

Cancer patients for radiotherapy are mostly elderly who have existing chronic medical conditions, such as heart disease and lung disease, and compromised immune systems, making them at higher risk of COVID‐19 infection. In addition, radiotherapy has unique characteristics, including heavy use of large medical equipment (CT, MRI, linac, etc.) and personalized medical devices (immobilization, respiratory management, dose measurement, etc.), and frequent interactions between staff. In order to reduce the risk of COVID‐19 infection during radiotherapy procedures, it is important for radiation oncology departments to develop and implement strict yet appropriate prevention and control measures to ensure effective treatment and care to patients during the challenging time.

In this letter, we will share our experience in COVID‐19 infection control in radiotherapy settings and provide recommended measures in potentially affected areas from a medical physics perspective. The Department of Radiation Oncology at Henan Cancer Hospital in China has eight linear accelerators, two CT simulators, and two brachytherapy high‐dose rate afterloaders. Departmental staffing includes 54 radiation oncologists, 26 medical physicists, and 45 radiation therapists. We treat about 1,000 patients per day before and about 600 people per day during the epidemic outbreak.

Since 25 January 2020, the beginning of the epidemic in China, our department has implemented strict infection prevention and control measures, including systematic training of COVID‐19 and SARS‐CoV‐2, strict implementation of protection regulations, standard office sterilization, wearing personal protective equipment (PPE), measuring body temperature every day, splitting work force, and adjusting work load, etc. The details of these measures are described below:

1. Access control at the entrance of the radiotherapy area
  - Temperature measurement is mandatory at the access control point.
  - In addition to basic medical clothing, medical caps, goggles, medical surgical masks, and disposable latex gloves must be worn by those who are responsible for disinfection and temperature measurement of patients and family members.
  - Check epidemic history and identity certificate of radiotherapy patients and their family members (specially tailored identifications by the hospital to patients and their family members during this special period, such as patient bracelets and accompanying cards).
2. Strict control of treatment time and treatment interval to prevent patients and their family members from interacting in the treatment area
3. Prevention and control of staff in the radiotherapy room
  - Wear qualified medical surgical masks or N95 grade masks, replace masks at the prescribed frequencies, check whether the masks worn by patients and their family members meet requirements (prevention of droplet transmission), and ask patients’ about their epidemiological history.
  - Wear disposable gloves (anti‐contact transmission).
  - Wear goggles (to prevent contact transmission).
  - Instruct patients and their family members not to touch door handles and devices in the treatment room (to prevent contact transmission), except for immobilization devices.
  - Provide disposable shoe covers and disposable gloves to patients and their family members (to prevent contact transmission).
  - Maintain distancing (no less than 1 meter) between all involved staff and patients and their family members.
  - Wash hands thoroughly after treatment (hand hygiene).
4. Disinfection in simulation and treatment rooms
  - Clean and disinfect equipment (CT simulator, linac, and operation table) twice a day using 75% alcohol wipe or spray, or 300‐500 mg/L chlorine disinfectant wipe or spray.
  - Disinfect floor using 300‐500 mg/L chlorine at least twice a day. When there are visible infection, first use disposable absorbent materials to completely remove the infection and then disinfect.
  - Disinfect door handles, buttons, treatment beds, tables and chairs, floors, and positioning devices using alcohol spread or any disinfectant.
  - Spray and wash the chlorine‐containing disinfectant using clean water (spray with disinfectant for at least 30 minutes before wiping with water).
  - Use ultraviolet light to disinfect room (no less than 1 hour, without people) for air disinfection.
5. Precautions for treatment planning
  - Wear and remove masks properly. If you go to the treatment room, wear disposable gloves, protective caps, and goggles.
  - Strictly implement hand hygiene, such as hand sanitizer, using flowing water, and washing hands after contacting patients.
  - Strictly implement office disinfection measures twice a day. To perform office disinfection, wear disposable gloves and spray 300‐500 mg/L of chlorine‐containing disinfectant to the floor, tabletop, chairs, cabinet doors, printers, curtain handles, and doors. After 30 minutes, clean the floor with water and wipe all furniture with clean water. Use 75% alcohol wiping or spray to disinfect the mouse, keyboard, and phone.
6. Precautions for attending face‐to‐face meeting. Wear a mask all the time, keep a sufficient distance from other meeting participants, open windows for better ventilation, and minimize the meeting time.

In summary, we have successfully implemented infection prevention and control measures in a radiation therapy department treating over 600 patients during the COVID‐19 outbreak in China. So far, none of our staff, patients, and their family members has been confirmed to have COVID‐19. Our experience could be of help to the medical physics community, especially to those who are currently in epidemic centers battling with the devastating virus.

## CONFLICT OF INTEREST

None

